# Draft genome sequences of *Passalora arachidicola* and *Nothopassalora personata*, causal agents of early and late leaf spots of peanut

**DOI:** 10.1128/mra.00153-26

**Published:** 2026-06-11

**Authors:** Kiersten R. Fullem, Wael Elwakil, Kristin A. Beckham, José C. Huguet-Tapia, Jeffrey A. Rollins, Nicholas S. Dufault

**Affiliations:** 1 Department of Plant Pathology, University of Florida 316814, Gainesville, Florida, USA; 2 University of Florida, UF/IFAS Extension, Hillsborough County, Florida, USA; University of California Riverside, Riverside, California, USA

**Keywords:** peanuts, groundnuts, fungi, leaf spot

## Abstract

We present draft genome sequences of the peanut leaf spot pathogens *Passalora arachidicola* isolate Ca1604 and *Nothopassalora personata* isolate Cp1602 that were generated using PacBio sequencing. The assemblies comprise 194 and 571 contigs with total lengths of approximately 37.75 and 49.04 Mb, respectively.

## ANNOUNCEMENT

Early and late leaf spots of peanut caused by *Passalora arachidicola* (syn. *Cercospora arachidicola*; teleomorph = *Mycosphaerella arachidis*) and *Nothopassalora personata* (syn. *Cercosporidium personatum*; teleomorph = *Mycosphaerella berkeleyi*), respectively, are among the most common foliar diseases of peanut worldwide and can cause severe yield loss (1). Despite their significance, there are currently very few publicly available genomes for these pathogens (2–4). To facilitate future studies on peanut leaf spot pathogen genetics, we have produced one high-quality draft genome assembly of each species.

Peanut foliage symptomatic for early or late leaf spot was collected in 2016 from a fungicide efficacy trial conducted at the University of Florida’s Plant Science Research and Education Unit in Citra, Florida. Conidia recovered from characteristic foliar lesions were observed using compound microscopy and confirmed as *P. arachidicola* or *N. personata* based on morphology. For both pathogens, a foliar lesion with abundant sporulation and no visible contaminants was selected and flooded five to 10 times with 12–14 µL of 0.005% Tween 20 (Acros Organics, Geel, Belgium). The resulting spore suspension was applied to a firm water agar plate amended with chloramphenicol, streptomycin, and tetracycline (all 50 µg/mL) using a sterile cell spreader. Plates were then sealed with parafilm and incubated for 16–24 h at room temperature. After incubation, well-isolated and germinating conidia were identified and excised from the media using a microblade. Single conidia were transferred to plates of antibiotic-amended potato dextrose agar (Difco PDA 25.6g/L; *P. arachidicola*) or oatmeal + yeast agar (Difco oatmeal agar 75.52g + Bacto yeast extract 2g/L; *N. personata*). After 3 weeks of incubation at 26°C under a 12 h photoperiod, the most vigorous and non-contaminated single-spore isolate of each species was selected for whole-genome sequencing.

These isolates, Ca1604 (*P. arachidicola*) and Cp1602 (*N. personata*), were then plated on peanut hull agar (350 g peanut hulls + 20 g granulated agar/L) and incubated for 6 and 12 months, respectively, at 26°C under a 12 h photoperiod. A sterile microscopy needle and forceps were used to harvest an area approximately 3–4 mm in diameter of pathogen biomass from the culture plates and remove media particles. Tissue was then placed into grinding tubes containing glass beads, frozen with liquid nitrogen, and ground into a powder using a bead-beater. Total genomic DNA was extracted using the OmniPrep for Fungus Kit (G-Biosciences, St. Louis, MO) and sent to the UF Interdisciplinary Center for Biotechnology Research for library preparation and whole-genome sequencing using the PacBio Sequel system (Pacific Biosciences, Menlo Park, CA). Resultant raw reads were assembled and polished using the Flye assembly pipeline (v. 2.9.3) using the argument “--pacbio-raw” (5). Each assembly’s quality and completeness were assessed using Quast (v. 5.2.0) with the argument “--fungi”; and BUSCO (v. 5.3.0) using the dataset fungi_odb10 (6, 7). Default parameters were used, except where otherwise noted. Assembly statistics are listed in Table 1.

**TABLE 1:** Genome information

Isolate	Ca1604	Cp1602
Species	*Passalora arachidicola*	*Nothopassalora personata*
Genome size (Mb)	37.75	49.04
GC content	50.96%	49.27%
# of contigs	194	571
# of reads	2,929,146	1,739,378
Read N50	9,734	11,790
Coverage	554×	228×
N50	629,177	300,088
N90	117,528	68,584
L50	19	44
L90	79	173
BUSCO completeness score	99.5%	99.2%

## Data Availability

This Whole-Genome Shotgun project has been deposited in NCBI under accession numbers GCA_978835725.1 and GCA_978805995.1. Raw reads are available under accession numbers SRX31671777 and SRX31671778.
